# Disintegrating Brain Networks: from Syndromes to Molecular Nexopathies

**DOI:** 10.1016/j.neuron.2012.03.006

**Published:** 2012-03-22

**Authors:** Jason D. Warren, Jonathan D. Rohrer, John Hardy

**Affiliations:** 1 Dementia Research Centre, Department of Neurodegenerative Disease, University College London Institute of Neurology, London WC1N 3BG, UK; 2 Reta Lilla Weston Laboratories and Departments of Molecular Neuroscience, University College London Institute of Neurology, London WC1N 3BG, UK

## Abstract

In this issue of *Neuron*, Raj et al. (2012) and Zhou et al. (2012) use graph theory to suggest that neurodegenerative diseases spread diffusively via intrinsic brain networks. These studies provide a powerful model for understanding and predicting disease-specific profiles of neurodegeneration.

## Main Text

Neurodegenerative brain diseases are collectively characterized by two core features: abnormal protein deposition and distinctive profiles of damage across the brain and over time (Frisoni et al., 2010; Rohrer et al., 2011). If we understood in detail how proteinopathies translate to clinical phenotypes, we might anticipate and perhaps prevent the devastating impact of these diseases. While we have recognized for some time that spatiotemporal brain atrophy profiles track neuropathological patterns of disease evolution (Frisoni et al., 2010), we have lacked a principled framework for understanding and predicting the profiles observed. The brain is composed of neural networks and graph theory provides a methodology for representing and analyzing those networks (Bullmore and Sporns, 2009). Work in animal models has demonstrated a correspondence between mathematically derived network characteristics and the hierarchical and distributed architectures of neuroanatomy (Modha and Singh, 2010). Network-level analysis is an ideal approach to understanding neurodegenerative diseases, due both to the fundamentally coherent and distributed nature of the underlying pathological processes and the failure of conventional approaches to adequately explain the distinctive phenomenology of these diseases. However, the potential clinical value of network-based approaches remains largely unrealized.

Two papers in this issue of *Neuron* (Raj et al., 2012; Zhou et al., 2012) take us further toward this goal, by applying the methods of graph theory to quantify and predict network disintegration in a range of neurodegenerative diseases. These papers capitalize on two key recent insights: the expression of neurodegeneration within specific, distributed, intrinsic brain networks (Zhou et al., 2010) and the propensity of culprit proteins to “template” further protein aggregation and spread of disease along neural pathways (Hardy, 2005; de Calignon et al., 2012). Raj et al. (2012) model network diffusion based on tractography data in the healthy brain and derive robust spatial eigenmodes that correspond closely to atrophy profiles observed in Alzheimer's disease and frontotemporal dementia; their model makes no prior assumptions about selective neuronal vulnerabilities or protein-specific factors. Zhou et al. (2012) show that common neurodegeneration syndromes seed distinctive connectivity structures derived using task-free fMRI in the healthy brain: their data suggest that the neurodegenerative process spreads primarily between neurons according to the functional proximity of specific brain regions acting as critical hub-like “epicenters,” rather than various alternative candidate mechanisms. Both papers agree that transsynaptic diffusion plays a core role in the spread of neurodegenerative pathologies, and together they provide a succinct framework for characterizing network disintegration in these diseases.

**Figure 1:**
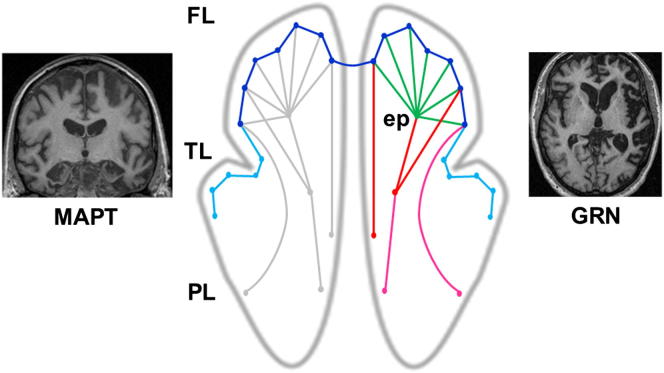
From Syndromes to Molecular Nexopathies The figure attempts to reconcile the transneuronal model of network degeneration suggested by the data of Raj et al. (2012) and Zhou et al. (2012) with empirical data on distinctive brain atrophy profiles associated with specific genetic proteinopathies (e.g., Rohrer et al., 2011). In the central brain schematic, circles represent local network elements and lines represent transneuronal anatomical connections (these need not correspond closely to the “edges” of graph theoretical representations: Zhou et al., 2012). The effects of proteinopathies (colored) on intrinsic network architecture (gray) in the frontal (FL), temporal (TL), and parietal (PL) lobes in each cerebral hemisphere are shown. The side panels show coronal (left) and axial (right) brain MRI sections representing typical atrophy profiles produced by mutations in the microtubule-associated protein tau gene (MAPT: relatively symmetrical, relatively localized anterior temporal and inferior frontal atrophy) and the progranulin gene (GRN: strongly asymmetric fronto-temporo-parietal atrophy). Behavioral variant frontotemporal dementia was the presenting syndrome in each case. Initial involvement of an epicenter (ep) in right orbitofrontal cortex propagates over common transneuronal pathways in the target network (green); we hypothesize that, in addition, MAPT mutations may preferentially damage shorter anatomical pathways within the target network (dark blue) and connected off-target network elements (light blue), while GRN mutations may preferentially damage longer intrahemispheric pathways within the target network (red) and connected off-target pathways (magenta) via a catastrophic reamplification process. This modulation of the basic diffusive template for network disintegration anticipates the distinctive atrophy profiles observed.

For clinical neurologists and molecular biologists, these elegant and sophisticated studies hold a strong intuitive appeal. That being said, the studies raise as many issues as they resolve. So where do we go from here? Viewed critically, these two studies are directed chiefly toward the “deep phenotyping” of neurodegenerative syndromes: the mapping between clinical profiles and permissive brain architectures. Less widely pursued has been the reverse mapping, from specific molecular pathologies via network breakdown to clinical disease; yet accurate prediction and tracking of molecular pathology from phenotype will be essential for the rational application of specific protein-targeting therapies. As Raj et al. (2012) and Zhou et al. (2012) point out, large-scale connectivity approaches have yet to settle such fundamental issues as the basis for initial targeting of particular brain regions by neurodegenerative pathologies, the role of protein-specific mechanisms in disease evolution and (perhaps most problematically of all) the typically wide variation in phenotypic expression among individuals with a particular molecular diagnosis. On the other hand, we already know that particular canonical syndromes can be produced by genetic mutations with radically different group-level brain atrophy profiles (Rohrer et al., 2011; see Figure 1). A complete network account of neurodegeneration will need to resolve such apparently paradoxical observations. In our view, progress is likely to depend on incorporating molecular pathological “minutiae” (Raj et al., 2012) into existing network models.

One way forward may be to assess patterns of network breakdown that segregate according to the morphology of network elements rather than networks in their neuroanatomical entirety. The idea that particular network components may be differentially vulnerable to neuropathological processes is implicit in the work of Zhou et al. (2012) and compatible with the results of Raj et al. (2012). Intrinsic brain connectivity and transsynaptic disease spread may be overarching principles, while within damaged networks, proteinopathies may operate via subsidiary mechanisms such as those delineated by Zhou et al. (2012) to produce specific profiles of network breakdown. Recent rapid progress in characterizing genetic and histopathological substrates of the frontotemporal dementias has enabled, for the first time, a more or less complete analysis of these diseases in molecular terms. Such analyses suggest that specific clinicoanatomical signatures of proteinopathies can be identified (Rohrer et al., 2010, 2011; Whitwell et al., 2012). In particular, there appears to be a partitioning between pathologies that produce largely symmetrical versus strongly asymmetrical cerebral degeneration and between pathologies that produce relatively localized versus widespread degeneration at a given disease stage. Although the common sporadic neurodegenerative diseases are the ultimate targets of molecular phenotyping approaches, rare genetic proteinopathies meanwhile constitute crucial test cases. Rather than mapping simply to a particular brain network, molecular specificity in these diseases may emerge as an interaction between large-scale configurational and local morphological factors (Rohrer et al., 2011). As acknowledged by Raj et al. (2012), complex systems may generate relatively simple outputs; with respect to disintegrating brain networks, one such simple dichotomy may apply to short- versus long-range connections. The “small-world” properties of brain networks (Bullmore and Sporns, 2009) lead us to expect that a short-range/long-range dichotomy should be functionally meaningful, and pathways might in turn show differential vulnerability to molecular lesions (we outline this as a testable hypothesis in Figure 1). “Short-range” and “long-range” here could be specified using anatomically grounded methods (Modha and Singh, 2010). Importantly, protein-specific mechanisms might also operate at the level of events that trigger the neurodegenerative cascade. For example, whereas initial targeting of entorhinal cortex in Alzheimer's disease may reflect locally enhanced beta-amyloid-precursor protein deposition during age-related neuronal resprouting (Roberts et al., 1993), progranulin-associated neurodegeneration may be triggered by an initial discrete stochastic (e.g., vascular hypoxic) event which becomes catastrophically amplified by failure of synaptic repair mechanisms (Piscopo et al., 2010).

As the work of Raj et al. (2012) and Zhou et al. (2012) shows, graph theory gives us a means to test specific hypotheses of brain network disintegration. We suggest that models of network degeneration will need to be informed by data from a wide variety of sources. For example, recent work on the selective vulnerability of network nodes to extinction under sociological and ecological events (Saavedra et al., 2011) may help generate models for the selective targeting of the epicenters identified by Zhou et al. (2012). In addition, the power of anatomical methods should not diminish the role of behavioral metrics: if appropriately generic computations can be measured, these are likely to inform our understanding of network organization. Models of human semantic processing, for example, make relatively specific predictions about permissive network architecture in semantic dementia (Lambon Ralph et al., 2010). Similar arguments favor the use of task-based as well as task-free fMRI to characterize damaged networks. Empirical longitudinal data on the evolution of network disintegration are sorely needed in order to determine the validity of predictive models (Raj et al., 2012). Finally, clinical neurologists and neuroradiologists, by identifying the sometimes counterintuitive (e.g., highly asymmetric) profiles thrown up by particular neurodegenerative diseases, can help inform and constrain the search for candidate mechanisms to explain such profiles. The power and beauty of data such as those presented by Raj et al. (2012) and Zhou et al. (2012) will be fully realized if we can move beyond syndromic disease maps to a taxonomy of protein-based network degenerations: “molecular nexopathies.”
